# *Fry* Is Required for Mammary Gland Development During Pregnant Periods and Affects the Morphology and Growth of Breast Cancer Cells

**DOI:** 10.3389/fonc.2019.01279

**Published:** 2019-11-21

**Authors:** Yan Liu, Xushen Chen, Zhihong Gong, Hao Zhang, Fan Fei, Xiaojiang Tang, Jie Wang, Peilin Xu, Helmut Zarbl, Xuefeng Ren

**Affiliations:** ^1^The Key Laboratory of Gene Engineering, Ministry of Education, Sun Yat-sen University, Guangzhou, China; ^2^Department of Epidemiology and Environmental Health, University at Buffalo, Buffalo, NY, United States; ^3^Department of Cancer Prevention and Control, Roswell Park Comprehensive Cancer Center, Buffalo, NY, United States; ^4^Guangdong Medical Laboratory Animal Center, Foshan, China; ^5^Department of Biostatistics and Bioinformatics, Roswell Park Comprehensive Cancer Center, Buffalo, NY, United States; ^6^School of Public Health, Rutgers, Environmental and Occupational Health Sciences Institute, The State University of New Jersey, Piscataway, NJ, United States

**Keywords:** *Fry* gene, conditional knockout mice, mammary gland development, Hippo/Yap signaling pathway, cancer cell growth

## Abstract

The *Fry* gene, located on chromosome 13, is an evolutionarily conserved large protein from yeast to human. Our previous study genetically linked the *Fry* gene with differential susceptibility to mammary carcinogenesis, but whether *Fry* affects mammary gland development and function, as well as the growth of breast cancer cells, is largely unknown. To define the consequences of *Fry* loss in the mammary glands, we have generated mice conditionally deficient of the *Fry* gene in the mammary glands using the Cre-loxP recombination system. We examined multiple phenotypes with male and female homozygous *Fry* conditional knockout mice (Mfry) and control mice (WT), including body weight, preliminary observations (health and neurological flexes), open field locomotion, sensory abilities, auditory threshold, and glucose metabolism. The loss of *Fry* in the mammary glands didn't cause a significant difference in these genotypes between Mfry and WT mice. However, our data showed that Fry was required during pregnancy, while it was functionally dispensable in virgin mammary gland development. Loss of Fry led to more lateral buds, and the lobuloalveoli were smaller and showed undistended morphology in mammary glands during late pregnancy. *in vitro* experiment, ectopic expression of FRY could alter the morphology and significantly suppress the growth and proliferation of the breast cancer cell lines, MDA-MB-231 (ER-/PR-/HER2-, Basal-like) and BT474 (ER+/PR+/HER2+, Luminal B). The following genome-wide transcriptomic analysis of these cells suggested that FRY interacted with protein kinases relevant signaling pathways and induced massive changes in gene expression, including the activation of the Hippo/Yap pathway. Together, our data suggest that the FRY is required for mammary glands developments during pregnant periods, and affects breast cancer cell growth and proliferation.

## Introduction

The furry (*Fry*) gene was originally identified in 2001 as a *Drosophila* gene involved in maintaining the integrity of cellular extensions during morphogenesis (1). It has 5–6 conserved regions, including FRY N-terminal domain (FND) consisting of HEAT/Armadillo-like repeats. Additionally, two leucine zipper motifs and coiled-coil motifs near the C-terminus have been found in FRY proteins of vertebrates (2, 3). Studies in lower eukaryotes showed that its orthologs (termed *Tao3p* in budding yeast, *Mor2p* in fission yeast, *Sax-2* in nematode, *Furry* in fruit fly and *Xfurry* in Xenopus) play crucial roles in various cellular processes, including cell polarization, division, morphogenesis, dendritic branching and tiling, spindle organization, and gene expression (2–11). Although the role of mammalian FRY remains to be determined, it has been suggested to play essential roles in chromosome alignment, spindle organization, and the stability of microtubules (MTs) during mitosis (12–14).

Recently, some studies have suggested a role of FRY in cancer development. For example, Kim et al. found that *EEF1DP3-FRY*, a recurrent fusion gene resulting in early truncation of the *Fry* gene was found in 6.7% (8/120) of the breast cancer samples analyzed (15). Studies showed that site-specific methylation of *Fry* could differentiate pancreatic ductal adenocarcinoma from other benign causes, which could be applied in pancreatic ductal adenocarcinoma diagnosis (16, 17). However, the role of FRY in tumor development and progression remains largely unknown. Our recent study genetically linked the *Fry* gene with differential susceptibility to mammary carcinogenesis (18). We thus are interested in understanding the etiology and cellular mechanisms of mammalian Fry in mammary development and tumorigenesis. Given that there was no appropriate animal model, we thus employed the MMTV cre-loxP system and generated mice model with the targeted deletion of the *Fry* gene in the mammary gland epithelial cells (Mfry). In this study, we examined multiple phenotypes between Mfry and control mice, focusing on the role of Fry in mammary gland development in different life stages. Moreover, our *in vitro* studies show that FRY affects morphology and suppresses the growth and proliferation of breast cancer cells, which is likely related to its interaction with protein kinases and associated signaling pathways.

## Materials and Methods

### Animals

**Figure 1:**
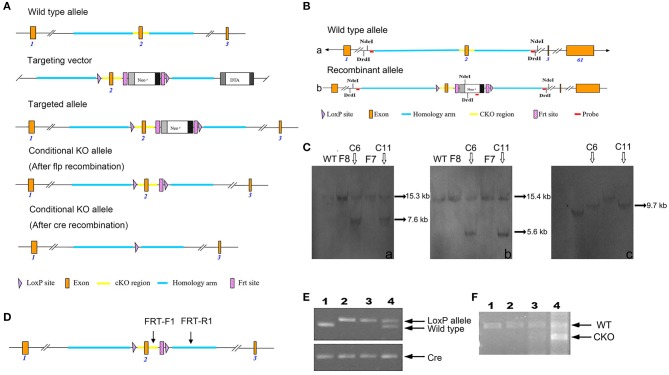
Generation of *Fry* CKO mice and genotyping. **(A)** Schematic explanation of the gene targeting strategy. **(B)** Southern blot strategy. Structure and restriction map of the wild-type *Fry* allele (a). Structure and restriction map of the recombinant *Fry* allele with insertions of two loxP sites in intron 1 and intron 2 and a neo gene flanked by Frt sits in intron 2 (b). **(C)** Southern blot analysis of the targeted clones. Six potential targeted clones were identified, four of them (F8, C6, F7, C11) were expanded and frozen, the Southern blot analysis was conducted by 5′-probe, 3′-probe, and Neo-probe. 5′probe. The genomic DNA of the potential clones F8, C6, F7, C11 was digested by NdeI and analyzed by Southern blot for a 15.3 kb band from wildtype allele and a 7.6 kb band from recombinant allele, C6 and C11 were positive (a). 3'probe. The genomic DNA of the potential clones F8, C6, F7, C11 was digested by DrdI and analyzed by Southern blot for a 15.4 kb band from wildtype allele and a 5.6 kb band from recombinant allele, C6 and C11 were positive (b). Neo probe. The genomic DNA of the potential clones F8, C6, F7, C11 were digested by NdeI and analyzed by Southern blot for a 9.7 kb band from recombinant allele, C6 and C11 were positive (c). **(D)** PCR genotyping strategy. Where conditional *Fry* KO allele, primers FRT-F1/FRT-R1 amplified a 719 bp product. Wild-type animals without the LoxP and Frt sites amplify a 583-bp PCR product using primers FRT-F1/FRT-R1. Arrows show positions of PCR primers used for detecting products of recombination. **(E)** PCR analysis of MMTV-cre-mediated recombination in Mfry and WT mice. Offspring with the desired genotypes were identified by PCR analysis of tail DNA. MMTV-cre mice were identified by Cre primers. The wild-type PCR product is 583 bp and the recombined is 719 bp. (1) Wild-type (*Fry*^+/+^, cre) mouse; (2, 3) Mfry (*Fry*^LoxP/LoxP^, cre) mouse; (4) Heterozygous (*Fry*^LoxP/+^, cre) mouse. **(F)** QPCR analysis the expression of FRY in the mammary gland of Mfry and WT mice. The wild-type PCR product is 782 bp and the recombined (exon 2 knockout) is 582 bp. (1, 2) Wild-type (*Fry*^+/+^, cre) mouse; (3, 4) Mfry (*Fry*^LoxP/LoxP^, cre) mouse.

Through cooperation with Cyagen Biosciences Inc., we successfully generated *Fry* CKO mice by a cre-loxP recombinant system. The targeting strategy is shown in Figure 1A. In the targeted allele, 2 loxP sites flank exon 2, whereas downstream we see the positive neomycin (neo) selection marker flanked by 2 Frt sites. The targeting vector was electroporated into C57BL/6J embryonic stem (ES) cells. After a positive/negative selection of ES cells with G418, 190 (one 96-well plate) G418-resistant clones were picked. Clones that had successfully undergone homologous recombination with the *Fry*-targeting construct were identified by PCR and confirmed by Southern blot conducted by 5′-probe, 3′-probe, and Neo-probe (Figure 1B). The probe primers are as follows: 5′ Probe primers: 5′ Probe-F: TTTACCAGGACCACCTCTTATGTCC, 5′ Probe-R: TGATTAGCAGACAGGATTCTCACACC; 3′ Probe primers: 3′ Probe-F: CTCTCATTGTGTTTCCAACAAGTACG, 3′ Probe-R: GGTCAGACAAAAGAACCATCCTGG; Neo Probe primers: Neo Probe-F: CCTGAATGAACTGCAGGACGAGG, Neo Probe-R: AGCTCTTCAGCAATATCACGGGTAGC).

ES cell clones that were confirmed to have undergone homologous recombination were frozen in liquid nitrogen and used for blastocyst injection. The ES cells were injected into blastocysts to generate chimeras. Tail DNA PCR was also performed to check the targeted alleles. The neo cassette, flanked by FRT sites, was removed by crossing the CKO line with a transgenic mouse expressing flper recombinase. This generated mice with a floxed *Fry* allele (*Fry*^+/LoxP^). The male offspring that were heterozygous for the floxed allele (*Fry*^+/LoxP^) were saved for breeding to female mice that were heterozygous for the floxed allele (*Fry*^+/LoxP^). Twenty-five percent of offspring from this mating have two floxed alleles (*Fry* ^LoxP/LoxP^) and are used in later mating to generate the tissue-specific deletion of the *Fry* gene.

To generate mammary gland-specific *Fry* knockouts, we crossed the Tg (MMTV-cre) 4Mam/J, Line D mice express a transgene containing Cre recombinase with widespread expression (but not in oocytes) under control the mouse mammary tumor virus-long terminal repeat (MMTV-LTR) promoter (19, 20) with *Fry*^LoxP/LoxP^ mice. Using this method we generated heterozygous mice (*Fry*^+/LoxP^*, cre*). Male Fry^+/LoxP^ mice were crossed with female *Fry*^+/LoxP^ mice that contained one transgenic allele expressing Cre recombinase. We choose *Fry*^LoxP/LoxP^ *cre* (Mfry) as our *Fry* CKO mice and *Fry*^+/+^ *cre* (WT) as our control. The animals had free access to food and water. We analyzed and compared phenotypes between Mfry (nine males and nine females) and control WT (seven males and seven females) mice.

All animal protocols were approved by the Institutional Animal Care and Use Committee. Related animal experiments were performed at the AAALAC-accredited vivarium at the University at Buffalo.

### Genotyping

Offspring with the desired genotypes were identified by PCR analysis of tail DNA using primers from regions containing the *Fry* Frt sites (Mfry: 719 bp; WT: 583 bp). MMTV-cre mice were identified by cre primers. PCR amplification of *cre* results in a 377 bp DNA fragment. Total RNA extracts were prepared from the mouse mammary gland tissue. The reverse transcription products were used to amplify a fragment of FRY. The sense/antisense primers across exon 2. The PCR primers for tail DNA (FRT-F1: CTGGTCTATTCACCCAACCA, FRT-R1: CATAGTCTCCCAAATAGAGGGTCAC, Mfry: 719 bp, WT: 583 bp; Cre-F: TGCCACGACCAAGTGACAGCAATG, Cre-R: ACCAGAGACGGAAATCCATCGCTC, Cre: 377 bp). The PCR primers for cDNA from mammary glands (Mfry-F: GCAAATAAGAAGCCAGCCCT, Mfry-R: CAGGATGAAGGGGGATCTGTT. Mfry: 782 bp, WT: 582 bp). Thermocycling conditions were as follows: 94°C for 3 min, followed by 30 cycles of 94°C for 45 s, 55°C for 30 s, and 72°C for 90 s and finally an extension step at 10 min at 72°C.

### Cell Lines

The human breast cancer cell lines MDA-MB-231 and BT-474, as well as human embryonic kidney cells (HEK293), were obtained from the American Type Culture Collection (ATCC). Cell lines were cultured in the medium recommended by ATCC and received from Life Technologies Inc. (Grand Island, NY). The cell medium was supplemented with 10% fetal bovine serum (FBS; Sigma-Aldrich, St. Louis, MO) and 1% penicillin-streptomycin and incubated at 37°C in a fully humidified atmosphere containing 5% CO_2_. All cell lines were used at a low passage in our laboratory, tested for viability, morphology, and growth curve analysis on a regular basis, and tested negative for mycoplasma. Vectors construction and stable breast cancer cell lines establishment are described in Supplementary Data.

### Behavioral Analysis

We performed a comprehensive analysis of the phenotype of *Fry* CKO and their WT, including physical characteristics, behaviors, morphology, the acoustic startle response, hearing sensitivity, and glucose tolerance. We also assessed the fourth mammary gland development at different stages using whole-mount preparations and hematoxylin and eosin (H&E) staining.

### Body Weight

The body weight test measured the weight of the mouse in a time series between 9 and 16 weeks.

### Mammary Gland Whole-Mount Preparation, Staining, and Analyses

Mammary gland whole-mount staining was performed following the standard procedure. In brief, the fourth inguinal mammary gland fat pads of WT mice were excised at virgin 4 weeks (*n* = 4), 8 weeks (*n* = 4), 12 weeks (*n* = 4), pregnancy 7 days (*n* = 3), pregnancy 14 days (*n* = 3), pregnancy 18 days (*n* = 3), lactation 8 days (*n* = 3), involution 6 days (*n* = 3), and involution 2 months (*n* = 4). Mfry mammary glands were excised at virgin 4 weeks (*n* = 4), 8 weeks (*n* = 4), 12 weeks (*n* = 3), pregnancy 7 days (*n* = 3), pregnancy 14 days (*n* = 4), pregnancy 18 days (*n* = 4), lactation 8 days (*n* = 3), involution 6 days (*n* = 3), and involution 2 months (*n* = 3). Tissues were then mounted on glass slides and fixed in Carnoy's fixative (75% ethanol, 25% glacial acetic acid) overnight at room temperature. The fixed glands were washed in 70% ethanol for 15 min and rinsed in distilled water for 10 min (twice). The mammary glands were stained with carmine red (1 g carmine and 2.5 g aluminum potassium sulfate in 500 mL water) overnight at 4°C and were then dehydrated progressively in 70%−95%−100% ethanol, cleared in xylene overnight at room temperature. Slides were mounted with Permount (Thermo Fisher Scientific). Mammary gland whole mounts were photographed using an inverted microscope (Zeiss Axio Observer).

### Tissue Preparation for Histological Analysis

For H&E staining, a portion of the third mammary gland was fixed in 4% paraformaldehyde in PBS at 4°C overnight. Fixed tissue was embedded in paraffin wax. For histological analysis, 6 μm sections were cut and stained with H&E.

### Cell Immunofluorescence Staining

Immunofluorescence was used to assess the *in-situ* expression of the FRY. MDA-MB-231 related cells were cultured on coverslips in DMEM medium with 10% FBS and penicillin/streptomycin at 37°C with 5% CO_2_ for 24 h. The cells were briefly washed in PBS, fixed with 4% formaldehyde for 30 min, permeabilized with 0.5% Triton X-100 in PBS for 10 min and incubated in blocking buffer (10% goat serum, 0.2% Triton X-100, 0.05% Tween-20 in PBS, pH 7.4) for 1 h at room temperature. The cells were then incubated with primary antibodies diluted in blocking buffer overnight at 4°C. After rinsing, the primary antibodies were detected with Alexa Fluor® 594 conjugated goat anti-mouse IgG (Cell Signaling Technology) or Alexa Fluor® 555 conjugated goat anti-rabbit IgG (Cell Signaling Technology) secondary antibodies diluted at 1:500 in blocking buffer for 1 h at room temperature. Coverslips were mounted using ProLong® Diamond Antifade Mountant with DAPI (Life Technologies) and fluorescence images were visualized and captured with a ZEISS Axio Observer inverted fluorescence microscope (ZEISS, Jena, Germany).

Primary antibodies and dilution used in applications include the following: mouse anti-FRY (2F2-D8-E3-G3) antibody (RayBiotech, 1:100), mouse monoclonal anti-β-Actin antibody (Sigma-Aldrich, 1:2,000).

### Cell Counting Assay (MTT), BrdU Cell Proliferation Assay

MTT assay was performed as described in a previous study (21). BrdU cell proliferation assay (2750, Millipore Sigma) was performed according to the manufacturer's instructions.

### RNA-Seq Analysis

Two repeats for parental, v-ctrl and FRY-expressing MDA-MB-231 cells were subjected to RNA-Seq analysis. The extraction of total cellular RNA from cells was carried out using TRIzol Reagent (Thermo Fisher Scientific) and purified with an RNeasy Kit (Qiagen). Subsequent RNA preparation steps were carried out at the Genomics and Bioinformatics Core Facility at the University at Buffalo. The RNA quality was assessed by agarose gel electrophoresis, spectrophotometry, and a BioAnalyzer (Agilent). The samples were then used to generate sequencing libraries with a TruSeq RNA Sample Prep Kit (Illumina) and sequenced on an Illumina HiSeq 2500 sequencer following the manufacturer's instructions. STAR (version 2.4.2a) was used to align raw sequencing reads to the human reference genome (hg19) and GENCODE annotation (version 19). We used RSEM (version 1.2.23) to measure gene and transcript abundances in Transcripts per Million (TPM); then the TPM was upper quartile normalized across all the samples. The genes with TPM larger than 1 in at least one sample were kept for the downstream analysis. The mapping statistics were summarized in supplemental data (Supplementary Table 1). We performed PANTHER Gene-Ontology analysis and Ingenuity® Pathway Analysis (IPA) on the list of genes differentially expressed between cells expressing FRY vs. v-ctrl cells (Supplementary Materials).

### Transfection of MDA-MB-231 Expressing FRY With siRNA Directed Against LATS1

Dicer-Substrate siRNA (DsiRNA) specific for LAST1 (5′-CCCAUAUAAUUAUCCGAAGCCUATT-3′ and 5′-AUGGGUAUAUUAAUAGGCUUCGGAUAA-3′) and Universal Scrambled Negative Control DsiRNA were ordered from IDT. MDA-MB-231 expressing *FRY* cells were transfected with 50 nM of DsiRNA-NS or DsiRNA-*LATS1* for 24 h using Lipofectamine® 2000 (Life Technologies, Grand Island, NY) following the manufacturer's protocol.

### Data Analysis and Statistics

The results from the open field were analyzed with two-way ANOVA followed by pairwise comparison. Prepulse inhibition, acoustic startle response, intraperitoneal glucose tolerance test, and body weight were analyzed by three-way ANOVA. *P* < 0.05 were considered as statistically significant. The statistical analysis was done with the SPSS (version 25.0) software. The student's *t*-test was used to compare the difference between cells expressing *FRY* vs. v-ctrl cells. Western blot data represent at least three experiments. Data represent the mean ± SD of the averages of at least three independent experiments. Significance for each experiment was noted in corresponding figure legends (^*^P < 0.05; ^**^P < 0.01; and ^***^P < 0.001).

## Results

### Phenotypic Analysis of Mfry Mice

The summarized strategy of generating *Fry* CKO mice by insertion of two loxP sites between exon 1 and 3 is illustrated in Figure 1A. The deletion of exon 2 resulted in the loss of function of the *Fry* gene by generating a frameshift from downstream exons. Following excision of exons 2, a stop codon (TGA) was generated in advance. This will lead to a truncated expression of FRY (39 aa). Six homologous recombinants ES cell clones were identified by genomic PCR (data not shown). The Southern blot analysis was conducted by 5′-probe, 3′-probe and Neo-probe (Figure 1B), which confirmed that there were two correctly targeted clones (Figure 1C). The ES cells were injected into blastocysts to generate chimeras. The mice were crossed and genomic DNA was isolated from mouse tail biopsies and genotyped by PCR. The sites of PCR primers for tail DNA were shown in Figure 1D. The insertions of loxP sites in mice carrying *Fry* conditional allele result in a 719 bp fragment and the WT mice has a 583 bp fragment (Figure 1E). The expression of FRY in mammary glands was confirmed by RT-PCR. Because the *cre* transgene was active in mammary epithelium and not in the stroma and adipocyte, *Fry* CKO mammary gland had two bands, including the WT FRY (782 bp) and the exon 2 deleted FRY (582 bp). The control mammary gland only had WT FRY (782 bp) (Figure 1F).

Overall, *Fry* CKO mice are healthy and fertile with normal longevity. We counted eight pairs of WT and Mfry offspring and six pairs of heterozygous mice (*Fry*^+/LoxP^, cre) offspring. The data were shown in Table 1. The Mfry group had a normal number of pups and sex ratios compared to WT group (*P* > 0.5). Mfry mammary gland was functional and dams could support their litters. Even after several pregnancies, these mice were able to raise their litters (data not shown).

**Table 1:** Number of live born pups and weaned rate mating from parents of different genotypes.

**Parents genotype**	**Live born pups/litter**	**Weaned pups/litter**		**Weaned rate (%)**
		**Male**	**Female**	
${Fry}_{,}^{\text{LoxP}/\text{LoxP}}$ *cre* (8 pairs)	8.75 ± 1.85	4.12 ± 1.45	4.25 ± 0.97	95.2
${Fry}_{,}^{+/+}$ *cre* (8 pairs)	9 ± 2.12	4.5 ± 1.32	3.75 ± 1.09	91.7
${Fry}_{,}^{+/\text{LoxP}}$ *cre* (6 pairs)	7.83 ± 2.54	3.17 ± 1.21	4.67 ± 2.05	100

There were no noticeable differences in overall performance and physical characteristics (body length, tail length) between male and female Mfry and WT mice (Supplementary Figures 1A–C). The maximal muscle strength of forelimbs and combined forelimbs and hind limbs were not different between Mfry mice and the control (Supplementary Figure 1D). The Mfry mice also have a normal hearing and acoustic startle response (Supplementary Figures 3A–C). In the open field, no genotypic differences were observed in locomotor activity or anxiety-like behavior between Mfry with WT mice (Supplementary Figures 2A–D).

**Figure 2:**
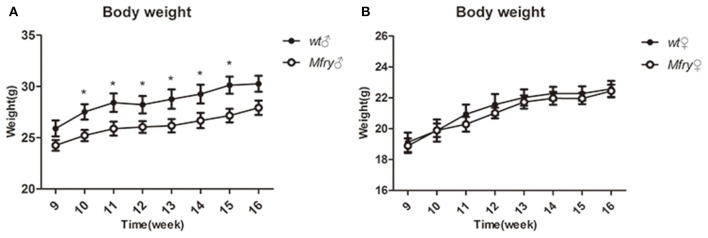
The growth curve of the mice. The body weight of the mice (*n* = 5 for male and female WT mice, *n* = 7 for male and female Mfry mice) from 9 weeks to 16 weeks was measured. **(A)** Male mice body weight between Mfry and WT. **(B)** Female mice body weight between Mfry and WT. Female mice show no difference between Mfry and WT mice. Male Mfry mice show the growth retardation when compare with WT male mice. Values are presented as means ± SEM. *P < 0.05. Mfry vs. WT.

There was an apparent gender effect of some phenotypic features of Mfry mice. For example, only the female Mfry mice showed differences in the open-field activity study with WT mice (Supplementary Figures 2B,C). However, in the intraperitoneal glucose tolerance test (IPGTT), it was the male Mfry mice, in which the glucose metabolism was faster than in WT mice after 30–60-min-glucose injections, and no differences were observed between female Mfry and WT mice (Supplementary Figure 4). Similarly, male Mfry mice had the body weight from 10 to 16 weeks significantly distinguished with male WT mice (Figure 2A), with no significant differences in body weight detected between female Mfry and WT mice (Figure 2B).

### The Deletion of *Fry* in the Mammary Gland Induced More Lateral Buds and Undistended Morphology in Pregnancy

**Figure 3:**
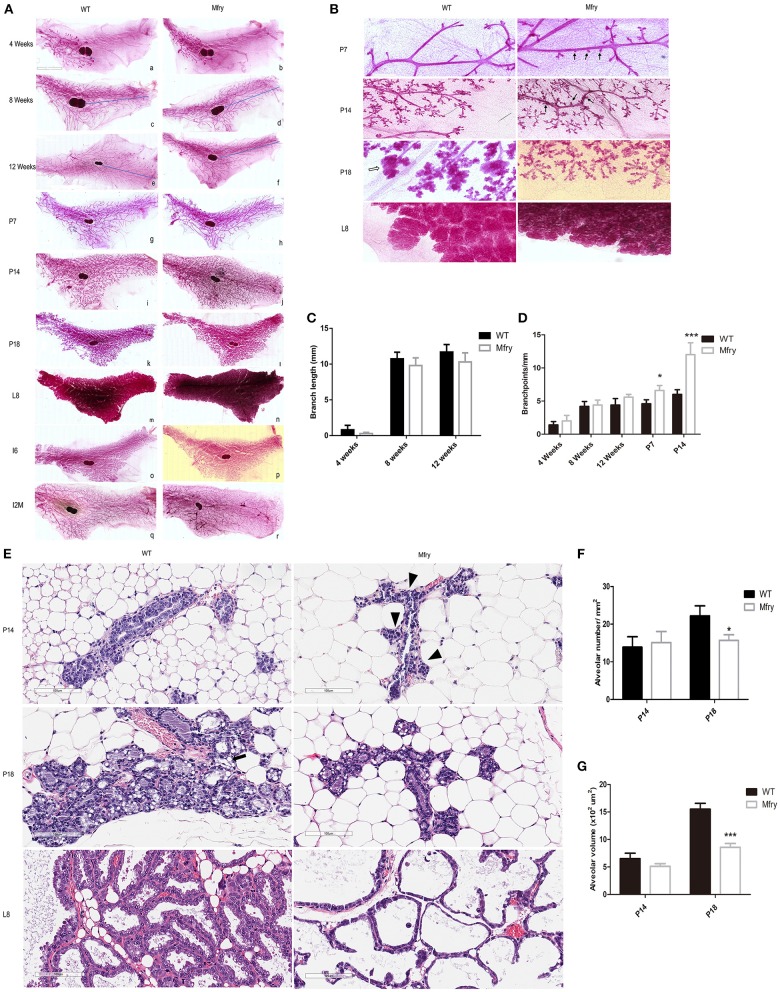
Impaired development in Mfry mammary glands. **(A,B)** Whole-mount staining of WT and Mfry mammary glands. Low-magnification view of mammary glands from several developmental phases, including ductal elongation in the virgin gland at 4 weeks (a,b), 8 weeks (c,d), and 12 weeks (e,f), proliferation, differentiation, and the formation of lobuloalveolar structures during pregnancy at 7 days (P7) (g,h), 14 days (P14) (i,j), and 18 days (P18) (k,l), lactation 8 days (L8) (m,n), involution 6 days (I6) (o,p), involution 2 months (I2M) (q,r). The blue line indicates the distance from the lymph node to the leading end bud. The appearance and ductal invasion in WT and Mfry mammary glands in virgin glands are similar (a–f). Bar, 2 mm. At pregnancy, Mfry glands display increased side branches (g–n). High-magnification view of P7, P14, P18, L8 glands in **(B)** more clearly shows that Mfry mammary glands display increased side branches (arrow), and also shows that the lobuloalveolar of Mfry mice glands are hypoplastic in pregnant 18 days compared to WT (white arrow). Bar, 400 μm. The structure of whole-mount mammary glands in lactation is not very clear because of the effect of milk (m,n). **(C,D)** Degree of ductal invasion was measured from the distal end of the lymph node to the furthest edge of ductal outgrowths, and the numbers of branch points per millimeter were determined by the Image J program. Data are mean ± SEM; n ≥ 3. *P* > 0.05, *t*-test. **(E)** H&E stains of WT, Mfry mammary glands at P14, P18, and L8 glands. Shown are representative images from H&E-stained tissue sections. Bar, 100 μm. The Mfry glands show more side branches (arrowhead), alveoli are smaller compared with control at P18. Also, note at P18 the presence of lipid droplets (black arrow) in control glands are far more than Mfry glands. **(F,G)** Comparisons of the numbers and areas of the alveoli among different genotypes at P14 and P18. The average numbers of alveoli per mm^2^ **(F)**. More than 30 alveoli were chosen to determine the average volume of the alveolus **(G)**. H&E-stained tissue sections were used for these analyses. Each bar is the average from more than three mice for each genotype. Student *t*-test was used for statistical analysis. Values are presented as means ± SEM. *P < 0.05, *** *P* < 0.001. Mfry vs. WT.

We performed whole-mount staining of mammary glands of Mfry and WT mice at various developmental stages, including pubescent (4 and 8 weeks old) and postpubertal virgin (12 weeks old), pregnancy (7, 14, and 18 days), lactation 8 days, involution 6 days, and involution 2 months (I2M). In pubescent and postpubertal virgin mice, when the primordial mammary tree is undergoing branching morphogenesis and invading into the fat pad, at 8 weeks, which marks the end of puberty in mice, the ductal extension nearly occupied all the fat pad (Figure 3A). Next, we analyzed *Fry* CKO mice mammary glands at pregnancy 7, 14, and 18 days, which contained significantly more lateral buds than those from WT glands during pregnancy at 7 and 14 days. At lactation 8 days, the mammary fat pads of Mfry and WT were filled with secretory alveolar structures. At involution 6 days, most of the Mfry and WT alveoli were involuted, and at involution 2 months the alveoli were involuted completely (Figures 3A,B). High-magnification views of P14 and P18 glands showed the smaller and undistended alveoli of *Fry* CKO glands (Figure 3B). To quantify the ductal elongation and branch points, the degree of ductal invasion was measured from the distal end of the lymph node to the furthest edge of ductal outgrowth, and the numbers of branch points per millimeter were determined by Image J program. The ductal elongation and ductal branch quantified no difference between Mfry and WT mice glands in virgin stages (Figures 3C,D). However, Mfry mammary glands showed a hyperbranched phenotype observed at pregnancy stages of development (Figures 3B,D).

In parallel, we prepared H&E staining of WT, *Fry* CKO mammary glands at P14, P18, and L8 stages for alveoli morphometrics (Figure 3E). Mfry mice had smaller alveoli and showed an undistended morphology at P14 and P18 and Mfry glands appeared less differentiated, and they contained tightly packed alveoli with fewer lipid droplets, a hallmark of mammary differentiation (Figure 3E). The average number of alveoli per square millimeter (mm^2^) in tissue sections and the alveolar volume at P14 and P18 were measured. The alveolar number and volume showed no difference at P14, while Mfry mice glands showed fewer alveoli per square millimeter and significantly smaller volume at P18 (Figures 3F,G).

### Ectopic Expression of FRY Changes Breast Cancer Cell Morphology and Suppresses the Growth and Proliferation of Breast Cancer Cell Lines

**Figure 4:**
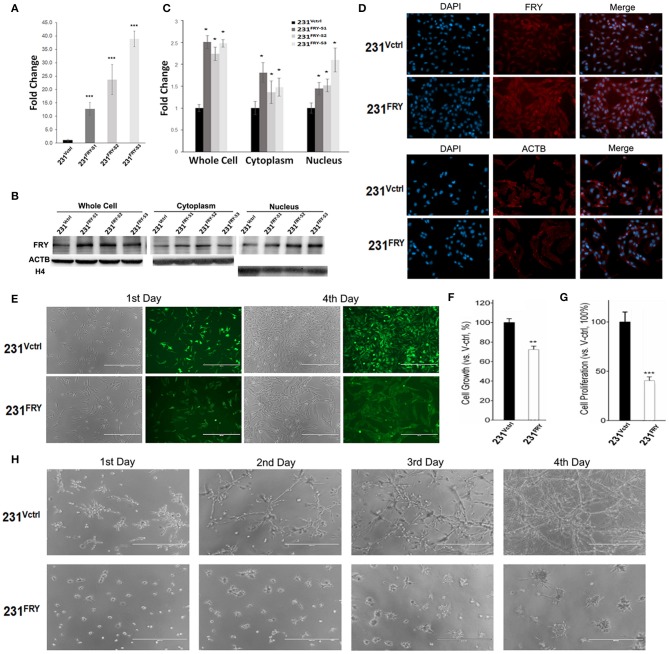
Ectopic expression of *Fry* changes breast cancer cell morphology and suppresses growth and proliferation of breast cancer cell lines. **(A)** FRY mRNA levels in established 231^Vctrl^ and 231^FRY^ cells. **(B,C)** A representative Western blot analysis shows the increased ectopic FRY protein levels in MDA-MB-231 human breast cancer cell line. Quantification of FRY level was performed by normalization to ACTB levels. **(D)** FRY and ACTB protein levels in 231^Vctrl^ and 231^FRY^ cells measured by cell immunofluorescence analysis **(**nuclei stained with DAPI). **(E)** Changed morphologies of MDA-MB-231 breast cancer cell lines in monolayer culture with the expression of *FRY*. **(F,G)** Cell growth and proliferation of 24 h were evaluated with MTT assay and BrdU assay. **(H)** The morphologies of 231^Vctrl^ and 231^FRY^ cells in three-dimensional Matrigel culture. Results are representative of three independent experiments. Bars represent the mean ± SEM of three independent experiments. Statistical significance: *P < 0.05; **P < 0.01; ***P < 0.001.

We established transfectants of MDA-MB-231 cells stably expressing the ectopic human *Fry* gene (named 231^FRY^). Cells harboring the empty vector were used as control (231^Vctrl^). Multiple clones of 231^FRY^ cells were isolated. We detected a significant increase in the FRY mRNA level in all clones of 231^FRY^ cells vs. in 231^Vctrl^ cells (Figure 4A). However, the FRY protein level was only increased moderately (about 2-fold) when analyzed by Western blot in all three clones of 231^FRY^ cells compared with 231^Vctrl^ cells (Figures 4B,C). The increase of the FRY protein level in 231^FRY^ cells was apparently more significant when it was measured by cell immunofluorescence analysis (Figure 4D). We also measured FRY protein level in the cytoplasm and nucleus, and the change of FRY levels in the cytoplasm and nucleus between 231^FRY^ cells and 231^Vctrl^ cells were largely comparable as measured using total cell lysates (Figures 4B,C). Because there was no significant difference in FRY protein levels among these clones, we picked the second clone of 231^FRY^ cells for the following research as its mRNA level was in the middle among three clones. 231^Vctrl^ cells exhibited an undifferentiated growth pattern and lacked contact inhibition in monolayer culture similar to the parental MDA-MB-231 cells. Ectopic expression of FRY alters the morphology of MDA-MB-231 cells, and 231^FRY^ exhibited a more organized, epithelial-like pattern of growth (Figure 4E). Ectopic expression of FRY significantly reduced cell growth and proliferation when assessed by MTT assay and BrdU incorporation assay, respectively (Figures 4F,G). In particular, the proliferation rate of 231^FRY^ was reduced by more than 60% (Figure 4G). When cultured with an overlay of Matrigel™ (three-dimensional cultures), we found that the 231^FRY^ cells grew much slower, displaying round/mass-like morphology (Figure 4H). In contrast, 231^Vctrl^ cells displayed aggressive and more disorganized stellate morphology (Figure 4H).

To determine whether FRY's cancer cell suppression effects would resonate across different breast cancer types, we constructed a similar breast cancer cell model based on BT474 human breast cancer cells (named 474^FRY^ and 474^Vctrl^ cells). BT474 human breast cancer cells belong to a less invasive Luminal B breast cancer type in comparison to highly aggressive MDA-MB-231 cells. Again, the FRY protein levels only moderately increased (about 1.5-fold), as analyzed by Western blot, but cell immunofluorescence analysis suggested a more significant increase in 474^FRY^ cells compared to parental BT474 and 474^Vctrl^ cells (Supplementary Figure 5). Morphological changes were less obvious, likely due to the growth character of BT474 cells in the culture (growth in clumps) (Supplementary Figure 6A). Both 474^FRY^ and 474^Vctrl^ cells grew slowly compared to MDA-MB-231 cells and showed a mass-like growth pattern, while 474^FRY^ cells seemed to grow with a more clearly defined cell boundary, as seen under the microscope, indicating the contact inhibition of growth. When cultured with an overlay of Matrigel™ (three-dimensional cultures), ectopic expression of FRY also apparently did not significantly alter the growth and morphology of BT474 cells (Supplementary Figure 6B); however, similar to MDA-MB-231, ectopic expression of FRY significantly reduced cell growth and proliferation assessed by MTT assay and BrdU incorporation assay, respectively (Supplementary Figures 6C,D).

### Elevated FRY Changes Gene Expression Globally, and Its Interaction With the Hippo/Yap Signaling Pathway Partially Contributes to FRY's Antitumor Functions

**Figure 5:**
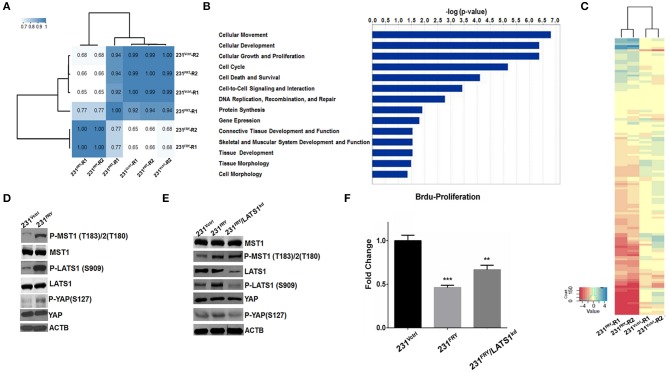
Elevated FRY changes gene expression globally, and its interaction with Hippo/Yap signaling pathway partially contributes to FRY's antitumor functions. **(A)** Clustering analysis of global gene expression profiles of 231^PRT^, 231^Vctrl^, and 231^FRY^ cells (two replicated for each cell line). **(B)** Top predicated molecular and cellular functions represented by differentially expressed genes between 231^Vctrl^ and 231^FRY^ cells, including cell movement, development, growth and proliferation, cell cycle, and cell death and survival. **(C)** Cluster heatmap analysis of genes regulated by Hippo/Yap kinase cascade. The majority of these genes were significantly downregulated in MDA-MB-231 cells expressing ectopic FRY. **(D)** Representative immunoblot images of total and phosphorylated MST1, LATS1 and YAP in 231^Vctrl^ and 231^FRY^ cells were shown. **(E)** MDA-MB-231 expressing ectopic *FRY* cells were transfected with non-specific siRNA (siRNA-NS), or siRNA-LATS1 for 24 h. Total and phosphorylated MST1, LATS1 and YAP were evaluated. **(F)** siRNA-LATS1 transfection in 231^FRY^ cells led to a compromised but not complete loss of FRY's effects in inhibiting proliferation of MDA-MB-231 cells. Results are representative of three independent experiments. Bars represent the mean ± SEM of three independent experiments. Statistical significance: **P < 0.01; ***P < 0.001.

Introducing *Fry* to MDA-MB-231 cells caused surprisingly massive changes in global gene expression, with 6340 genes whose expression changed at least 2-fold and at an FDR level of <0.01. As a result, genome-wide gene expression profiles were shifted, and unsupervised hierarchical clustering analysis of the global gene-expression profiles clustered the 231^FRY^ into a different clade and separated with 231^Vctrl^ cells and MDA-MB-231 parental cells (231^PRT^) (Figure 5A). We input all 6,340 genes into the IPA package and filtered it using breast cancer cell lines as target cells. A total of 4,206 genes was included in the bioinformatics IPA analysis. The IPA package's regulator-effects tool was then employed to examine the link between these changed molecular and cellular functions and upstream molecules, which helped us to understand the underlying mechanism and explain the differential patterns of expression observed between 231^FRY^ and 231^Vctrl^ cells. The IPA analyzed molecular and cellular functions that demonstrated the great changes, including cell movement, development, growth and proliferation, cell cycle, and cell death and survival (Figure 5B).

FRY has been suggested to physically interact with key molecules in the Hippo/Yap signaling pathway (1, 8, 12). A Heatmap analysis of RNA-Seq data clearly showed that the majority of Hippo/Yap pathway downstream genes were significantly downregulated in 231^FRY^ vs. in 231^Vctrl^ cells (Figure 5C, Supplementary Table 2) (22–27). This was consistent with the expected effects of the activation of the Hippo/Yap pathway. We thus examined whether enhanced FRY activates this signaling network and subsequently causes expression changes in downstream genes. We measured protein levels of key signaling molecules involved in the human Hippo/Yap signaling pathway, MST1 (serine/threonine kinase 4), LATS1 (large tumor suppressor kinase 1), and YAP (Yes-associated protein 1). Western blot analyses showed that ectopic FRY significantly increased the phosphorylation of MST1 and LATS1 and slightly increased the phosphorylation of YAP without changing the total levels of these proteins (Figure 5D). To further determine whether FRY's interaction with the Hippo/YAP signaling pathway contributes to the observed FRY's tumor suppressive function, we used a siRNA targeting the LATS1 mRNA and reduced endogenous LATS1 protein levels by about 85% in the 231^FRY^ cells (231^FRY/^LATS1^KD^) (Figure 5E). The LATS1 protein reduction lowered levels of phosphorylated LATS1 and YAP, whereas the total MST1 and YAP protein levels, as well as the phosphorylated MST1 level, remained comparable to 231^FRY^ cells (Figure 5E). The knockdown of *LATS1* increased the proliferation of 231^FRY/^LATS1^KD^ cells when compared to 231^FRY^ cells, but remained significantly lower than the 231^Vctrl^ cells (Figure 5F).

## Discussion

*Fry* was identified in our previous genetic linkage studies using inbred rat models of heritable mammary cancer to identify susceptibility genes and was associated with the resistance of the COP rat strain to N'-Methyl-N'-Nitrosourea (NMU) -induced mammary carcinogenesis (18). In this study, we created the first mouse model with the deficient *Fry* in mammary glands. We identified a novel role for Fry in regulating mammary gland development. The results presented so far suggested that Fry was dispensable in virgin mammary glands but was required during pregnancy. Loss of Fry during pregnancy increased the lateral buds and resulted in mammary epithelial cell differentiation defects. During late pregnancy, the lobuloalveolar structures were much bigger and distended in WT mammary glands compared to Mfry glands. In WT gland alveolar cells, it produced more lipid droplets, a hallmark of terminal mammary gland differentiation, suggesting that the loss of Fry results in the differentiation defects of mammary epithelial cells. However, we also noted that *Fry* CKO mice were able to secrete milk and did not cause a developmental delay of the offspring mice, suggesting that knocking out *Fry* in mammary glands could not prevent the ultimately differentiation of mammary epithelial cells and milk secretion.

In searching the literature, we found that our results mimic those reported for mice with the MMTV-rtTA driver mediated overexpression of YAP (28). YAP is a core protein in the Hippo pathway, which can be phosphorylated and lead to the activation of the Hippo-Yap signaling pathway. Hippo/Yap signaling and its regulated molecules play a central role in the growth of different tissues during development, regeneration, and tumorigenesis by modulating cell proliferation and contact inhibition (29, 30). *Yap* transgenic mouse models show that overexpression of YAP can result in mammary gland differentiation defects (24, 28, 31). YAP overexpression in mammary epithelium, like *Fry* knockout, did not affect mammary ducts in virgin glands. In pregnancy, however, the overexpression of YAP led mammary glands to fail to undergo terminal differentiation and showed an undescended morphology, a phenotype very similar to what we observed in Mfry mice (Figure 5B). Previous studies in lower eukaryotes showed that Fry proteins and nuclear Dbf2-related (NDR) family Ser/Thr kinases functioned in a common signaling pathway (12, 32, 33). The NDR protein-kinase family, which is at the heart of the Hippo/Yap kinase cascade pathway (18, 34, 35), was reported to directly interact with FRY that acted as an activator and scaffold protein (9, 25, 34, 36). It is known that the dysfunction of Hippo signaling may contribute to blocking the terminal differentiation of the mammary gland (31). It is possible that *Fry* deficiency can induce accumulation of un-phosphorylated YAP in nuclear and dysfunction of Hippo signaling, and ultimately results in the defect in alveolar differentiation upon *Fry* knockout similar to the effect observed in mice with overexpressed YAP. We thus measured total and phosphorylated levels of MST1, LATS1, and YAP protein in mammary glands from both Mfry and WT mice. However, the results hardly showed any significant differences in protein levels between Mfry and WT mice (data not shown). This could be because the proteins were extracted from total mammary glands with mixed cell types, but only mammary epithelial cells in Mfry mice had FRY deficiency, thus compromising the results. More delicate methods and better antibodies should help to determine the exact interaction between FRY and Hippo-Yap signaling pathways in our *Fry* mice model.

To explore the molecular function of FRY and further examine the interaction between FRY and Hippo-Yap signaling pathways, we established stable transfectants of the human *Fry* gene in two human breast cancer cell models, MDA-MB-231 (ER-/PR-/HER2-, Basal-like) and BT474 (ER+/PR+/HER2+, Luminal B). We showed that enhanced FRY significantly altered breast cancer cell morphology, growth and proliferation, suggesting that FRY may play a role in breast cancer development and progression.

The following genome-wide transcriptomic analysis demonstrated that introducing *Fry* to MDA-MB-231 cells could cause a global change of gene expression, especially with the moderate increase in FRY protein levels. Specifically, the data showed that ectopic FRY caused significant downregulation of Hippo/Yap regulated signaling molecules under the performed clustering analysis of the downstream targets of Hippo-Yap signaling based on their expression levels. Our following experiments confirmed that enhanced FRY had a significant effect on Hippo/Yap signaling pathways, resulting in increased levels of phosphorylated protein of key Hippo/Yap kinase, MST1, LATS1, and YAP. Knocking down *LATS1* partially reduced the effects of elevated FRY in inhibiting the growth and proliferation of breast cancer cells. Together, it suggests that FRY's function may be partially related to its interaction with Hippo/Yap signaling pathways. However, due to current experimental conditions, especially the available anti-*Fry* antibody, we could not determine whether FRY physically, directly or indirectly, interacts with Hippo/Yap kinases (e.g., MST1, LATS1, and YAP). Our lab has recently re-designed FRY overexpression systems with tags inserted after the C terminus of *Fry* gene, and we expect that this will enable us to perform co-immunoprecipitation or other methods to determine and study the interaction between FRY with these kinases and other protein kinases.

*Fry* is one of the largest genes in the human genome, with coding sequences of more than 10,000 base pairs, and is highly conserved at the amino acid level across species (35). Very recently, FRY was given the official name of FRY microtubule binding protein because studies done on mammalian cells suggested that FRY interacts with mitotic kinases (i.e., polo-like kinase-1, PLK-1) and is involved in the regulation of chromosome alignment and microtubule acetylation (13, 14). Although we expected that altering FRY levels would change the expression of some genes given previous reports between microtubule disruption and gene expression (37, 38), the global gene expression changes observed in 231^FRY^ cells could not be explained solely by FRY's interaction with microtubules. Both IPA and GO analyses of RNA-Seq data suggested that genes whose expression changed significantly were enriched in the activation of protein kinases, indicating the potential involvement of FRY in kinases mediated signaling pathways.

Despite these important results and observations, we are aware of the potential limitations of this study and the need for further studies and validation. In the initial phenotypic evaluation, there were no marked differences between Mfry and WT littermates in health, behavioral performance, physical characteristics (body length, tail length, muscle strength), rates of pup survival, and lifetime. The Mfry mice also have a normal hearing and acoustic startle response. However, we do observe some gender-specific differences; for example, in 10–16 weeks, male Mfry mice show growth retardation (*P* < 0.05–0.01). We have no specific explanation for these sex differences, and it is difficult to speculate about their relevance for *Fry's* function. The Cre recombinase is under the control of the mouse mammary tumor virus long terminal repeat (MMTV-LTR). Because the MMTV-LTR is active not only in mammary tissue but also in other secretory cell types and the hematopoietic system (19, 20), it is possible that the deletion of the *Fry* gene may affect not only mammary glands but also accidentally impact other mouse tissues. It is also possible that these phenotypes were “false-positive” observations or that these phenotypes were too subtle to detect using small numbers of mice (36). We are in the process of generating *Fry* knockout mice for all cell types. This should help us to clarify *Fry*'s impact on all body tissues in the near future. Our evaluation of breast cancer cells with enhanced FRY levels provided largely indirect data related to the potential tumor suppressing role of FRY. Similarly, the direct data are lacking to support the interaction between FRY and Hippo/Yap kinases. In the near future, when the new *in vitro* FRY overexpression system with tags is established, it will allow us to detect and study the interaction between FRY and protein kinases, which will help us to understand the mechanistic insights of how FRY affects signaling molecules and produces this massive effect on gene expression regulation and in anti-tumor functions. *In vivo*, we plan to isolate mammary epithelial cells (MECs) in different mouse mammary gland developmental stages, using quantitative phosphoproteomic and proteomic analysis to verify and identify protein kinase substrates and dynamic protein profiling of FRY.

## Data Availability Statement

RNA-Seq data sets reported here have been deposited in the Gene Expression Omnibus (GEO) with accession numbers GSE112910.

## Ethics Statement

The animal study was reviewed and approved by Association for Assessment and Accreditation of Laboratory Animal Care-accredited vivarium at the University at Buffalo.

## Author Contributions

YL, XC, and ZG designed and performed most of the experiments and helped to write the manuscript. HZh performed the initial research in cell construction and characterization. ZG and JW performed all statistical, bioinformatic analysis, and helped in manuscript development. XT and FF provided the anti-FRY antibody and did some animal experiments. PX critically read the manuscript. HZa led in the finding of the Fry gene, provided critical suggestions on project design. Overall project design and manuscript preparation was conducted by XR.

### Conflict of Interest

The authors declare that the research was conducted in the absence of any commercial or financial relationships that could be construed as a potential conflict of interest.
