# Supplementary material for: Fry Is Required for Mammary Gland Development During Pregnant Periods and Affects the Morphology and Growth of Breast Cancer Cells
Source: Front Oncol. 2019 Nov 21;9:1279. doi: 10.3389/fonc.2019.01279 (PMC6881260; doi:10.3389/fonc.2019.01279)
Supplement: Supplementary file 1 [file Data_Sheet_1.pdf]

Supplementary Materials

Supplementary Methods

SHIRPA

The SmithKline/Harwell/Imperial College/Royal Hospital/Phenotype Assessment (SHIRPA) was conceived as a multi-test battery. The purpose of the assessments is to examine mice for obvious physical characteristics, behaviors and morphological abnormalities.

Grip strength

The grip strength test was carried out with Bioseb G3 (Chaville, France). This test used to measure the neuromuscular function as maximal muscle strength of forelimbs and combined forelimbs and hind limbs. These are assessed by the grasping applied by the mouse on a grid that is connected to a sensor. Three trials are carried out in succession measuring forelimb-strength only, followed by three successive trials measuring the combined forelimb/hindlimb grip strength.

Open field

Animals were transported to the testing room (or ideally an antechamber) and left undisturbed for 30 minutes before the test. Testing is conducted during the light phase of the cycle with 1 hour gap from the light/dark change. Each mouse is placed in the middle of a peripheral zone of the arena facing the wall and allowed to explore freely the apparatus, with the experimenter out of the animal's sight. By using a video tracking system, the locomotor activity travel distance in the whole chamber as well as in the center area was monitored and using Topscan analysis software (CleverSys Inc., VA, USA) to analysis data.

Auditory brainstem response

Auditory brainstem response (ABR) test determines hearing sensitivity and other physiological parameters using evoked potential recordings in

anesthetized mice. ABR test was examined with RZ6 Z-Series Processor Bioacoustic System (Tucker Davis Technologies, USA) and analyzed with BioSig software. ABRs are recorded to clicks (10μs duration, positive transient) presented from 0-85 dB SPL in 5dB steps, presented 256 times at 42.6/sec. ABRs are recorded to the following frequencies and levels; 6kHz (0-85dB SPL), 12kHz (0-85dB SPL), 18kHz (0-85dB SPL), 24kHz (0-85dB SPL) and 30kHz (0-85dB SPL), presented in 5dB intervals. Tone pips are 5ms in duration, with a 1ms rise/fall time, presented 256 times at 42.6/sec (optional values). Tone stimuli are presented in decreasing frequency order for a particular sound level and from low to high stimulus level.

Prepulse inhibition

The acoustic startle response is characterized by an exaggerated flinching response to an unexpected strong auditory stimulus (pulse). This response can be attenuated when it is preceded by a weaker stimulus (pre-pulse) and is the principle underlying pre-pulse inhibition (PPI). PPI was examined with the SR-LAB Startle Response System (San Diego Instruments, San Diego, California, USA). During all testing, background was set at 65-dB white noise. The PPI test included a 5-min acclimation period, followed by 10 trial types comprising 20-ms pre-pulse, 80-ms interval, and presence or absence of 60-ms startle stimulus. Pre-pulse intense was set at background, 74, 82, or 90 dB, and the startle stimulus was 120 dB. Pure background or only startle was also included. The calibration of the load cell platform amplifier and the white noise tone was performed every time before the test. After the mouse was enclosed in the 3.8-cm diameter cylinder above the load platform, the PPI session began with 2 presentations of "pulse only" trial in 5 minutes, which were excluded from statistical analysis. Next, all the 10 types of trials were repeated 10 times in pseudorandom order. The inter-trial interval (ITI) was set 5 random numbers between 10 and 20 s. Maximal peak-to-peak amplitude was used to determine the ASR in the acoustic startle pulse and pre-pulse alone trials. Startle response was recorded every millisecond for 100 ms after the onset of startle, i.e. 60 ms during the startle plus 40 ms after the startle ended. The amount of pre-pulse inhibition (PPI) was calculated as a percentage score for each acoustic pre-pulse trial type: % PPI= 100 x (S – PPi_S)/S.

Intraperitoneal glucose tolerance test

We performed intraperitoneal glucose tolerance test (IPGTT) by challenging the mice with high dose of glucose (2 g/kg body weight). Fast mice overnight for approximately 16 hours by transferring mice to clean cages with no food or faeces in hopper or bottom of cage. During the experiment, we ensure that they have access to drinking water at all times. A small drop of blood (<5μl) is placed on the test strip of the blood glucose meter.

This is the baseline glucose level (t = 0). Then the mouse were inject intraperitoneally with the appropriate amount of glucose solution. The blood glucose levels are measured at 15, 30, 60 and 120 minutes (t = 15, t = 30, t = 60 and t = 120) after glucose injection.

Construction of piggybac-FRY transposon vectors

Human FRY cDNAs were amplified from HEK293 that carry the wild-type *FRY* allele (*FRY*). Double-stranded cDNA was prepared using a cDNA Amplification Kit (Life Technologies, Grand Island, NY). Then, briefly, the human *FRY* cDNA was cloned in four sections and then reconstructed into the TOPO pCR-XL vector (Life Technologies, Grand Island, NY), and subjected to DNA sequencing. Among multiple primers sets used, two modified primers, 5'-GCGCGCTAGCGCCACCATGGCCAGCCAGCAGGATTCGG-3' and 5'-GCACGGCTGCGGCCGCTCAGAGACTAGTGCCAGAA ACA-3', were used at the end of PCR amplification. The pCR-XL-TOPO- *FRY* vector was then excised using *Nhe*I/*Not*I restriction enzymes (New England Biolabs, Ipswich, MA) and subjected to gel purification. The nucleotides of the FRY gene containing NheI and NotI overhangs were annealed and ligated to a linearized piggybac vector (System Biosciences, Mountain View, CA) similarly digested with *Nhe*I and *Not*I enzymes. The constructed vectors were named *piggybac-FRY* transposon vector, respectively.

Establishment of stable breast cancer cell lines with human FRY expression using the piggybac transposon system

To generate breast cancer cells with a stabilized expression of human FRY genes, the cells were transfected with constructed piggybac-FRY (amplified from 293 cells and sequencing confirmed wild-type status) transposon vector or an empty vector counterpart. Briefly, the 0.5 μg piggybac-FRY transposon vector and the 0.2 μg Super piggybac transposase (System Biosciences) plasmid were used with 8 μl SBI's PureFection transfection reagent for one NucleoFection reaction. After 72 hours, the piggybac transposase activity was terminated, and cells were assessed by an AMG EVOS XL microscope (Advanced Microscopy Group, Bothell, WA). A green fluorescent marker yields a bright green fluorescence, permitting direct monitoring of delivery efficiency. Finally, the cell populations were sorted by the Becton Dickenson FACSAria II high-speed 10-color cell sorter (BD, Franklin Lakes, NJ), and cells with green fluorescence were purified. The breast cancer cells with stabilized expression of FRY were further selected with 1 μg/mL puromycin starting five days after transfection (Life Technologies, Grand Island, NY).

Western blot analysis

For immunoblot analysis, the cells were collected in cell lysis buffer (Cell Signaling, MA). Total protein was quantified using Coomassie protein assay reagent (Bio-Rad). An equal amount of protein (30 µg) was separated by SDS-PAGE and electrotransferred onto PVDF membrane. Immunocomplexes were visualized with enhanced chemiluminescence detection kits (Pierce, Rockford, IL). Total and phosphorylated MST1, LATS1, YAP and PLK1 antibodies were from Cell Signaling Tech. (Danvers, MA). Anti-β-actin antibody was purchased from Sigma-Aldrich (St. Louis, MO) and used as an endogenous control for equal loading. The monoclonal anti-FRY antibody was a custom synthesized product.

PANTHER Gene-Ontology analysis and IPA pathway analysis

The RNA-Seq application produced a list of differentially expressed genes between cells expressing FRY^{293} vs. v-ctrl cells. We conducted a Gene-Ontology analysis using the PANTHER classification system to perform the statistical over-representation test on these differentially altered genes (Mi et al., 2013). To further systematically determine canonical signaling pathways and molecular networks that the differentially expressed genes might involve, we performed a pathway/network enrichment analysis using the Ingenuity Pathway Analysis tool from Ingenuity Systems (Ingenuity Systems Inc.). We input all 6,340 genes into the Ingenuity® Pathway Analysis (IPA) package and filtered it using breast cancer cell lines as target cells. A total of 4,206 genes was included in the bioinformatics IPA analysis to assess pathways, biological function, and upstream activity (Knight et al., 2014). For canonical signaling pathway analysis, given a list of genes, a right-tailed Fisher's exact test was performed for the enrichment of these genes in its hand-curated canonical pathway database. Here, the p-value calculated for a pathway measures the probability of being randomly selected from all of the curated pathways. In our study, we used a cut-off of the corrected P-value less than 0.05 (or score > 1.30, here score = -log p) to define the significant pathways. For network enrichment, the differentially expressed genes were overlaid onto a global molecular network developed based on the Ingenuity Pathways Knowledge Base, in which functional relationships such as activation, chemical-protein interaction, expression, inhibition and regulation of binding were manually curated. Subnetworks of genes were then extracted from the global molecular network based on their connectivity using the algorithm developed by IPA. For each subnetwork, a likelihood score, which measures the probability of the differentially expressed genes being found in the same subnetwork by chance, was transformed from the p- values calculated by right-tailed Fisher's exact test. Additionally, the Ingenuity Pathway Analysis assigned the top three biological functions for each network is identified. The heatmaps for genes of interest were grouped by hieratical clustering using the R package.

Supplementary References:

Knight, J.M., Davidson, L.A., Herman, D., Martin, C.R., Goldsby, J.S., Ivanov, I.V., et al. (2014). Non-invasive analysis of intestinal development in preterm and term infants using RNA-Sequencing. *Sci Rep* 4**,** 5453. doi: 10.1038/srep05453

Mi, H., Muruganujan, A., Casagrande, J.T., and Thomas, P.D. (2013). Large-scale gene function analysis with the PANTHER classification system. *Nat Protoc* 8(8)**,** 1551. doi: 10.1038/nprot.2013.092

Supplementary Figures

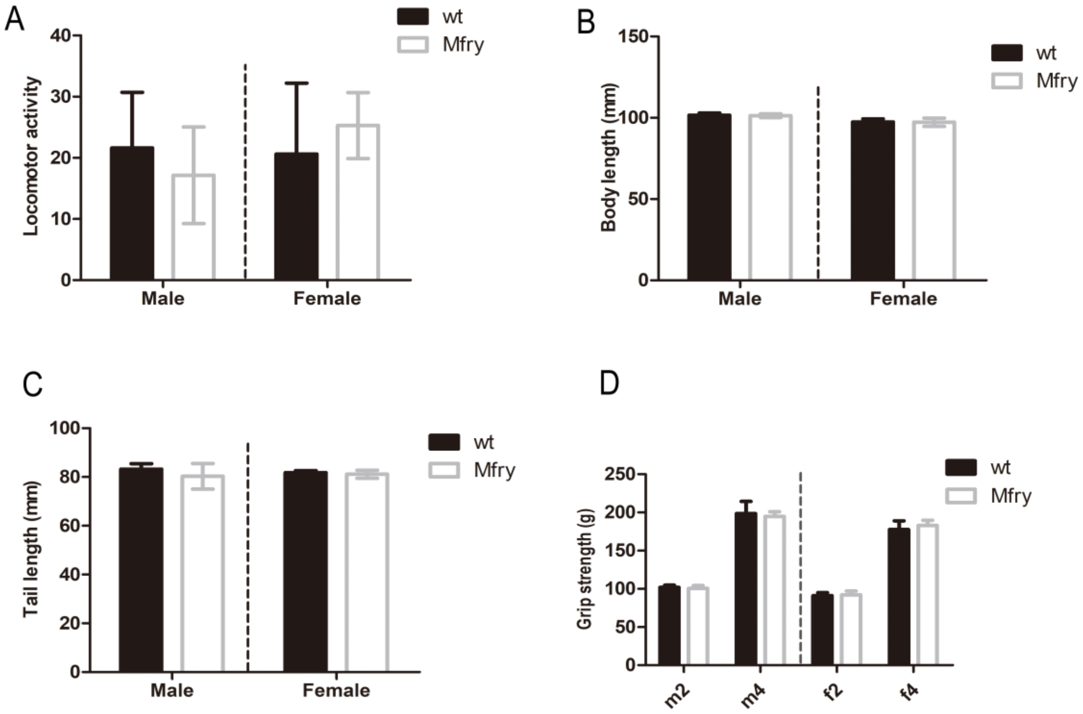

Supplementary Figures 1. Regular locomotor, physical characteristics and neuromuscular function appeared normal with no difference compared to WT mice (both sexes). 9 weeks old mice (n=5 for male and female WT mice, n=6 for male and female Mfry mice) were used. (**A-C**) SHIRPA. The physical characteristics, behaviors and morphological abnormalities were detected and showed no difference between KO mice and the control. Locomotor activity (A), body length (B), tail length (C). (**D**) Grip strength. The maximal muscle strength of forelimbs and combined forelimbs and hind limbs was no difference between KO mice and the control. m2, m4, f2, f4 respectively represent the grip strength of male forelimb, male fore and hind limb, female forelimb and female fore and hind limb. Data presented as mean ± SEM. Mfry versus WT.

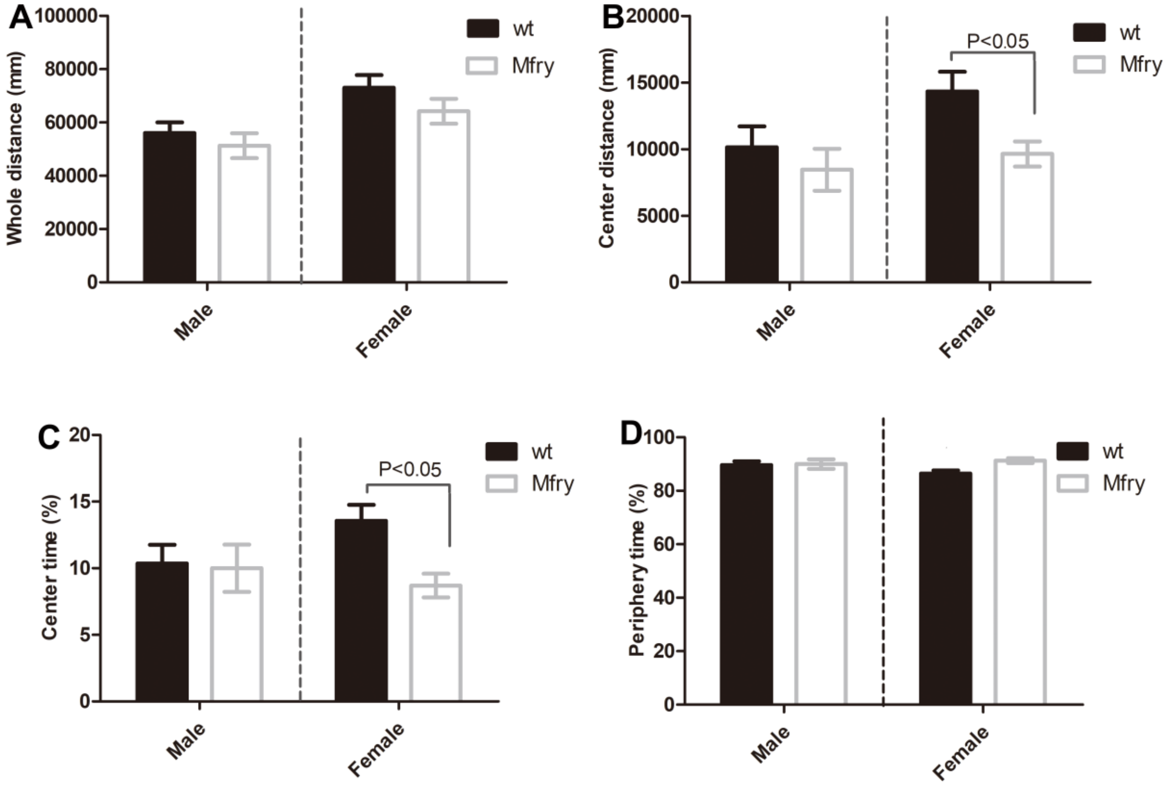

Supplementary Figures 2. (A-D) Female *Fry* KO mice show less activity in the open field test. WT and Mfry mice at 9 weeks old were placed in an open field arena and allowed to explore freely the apparatus. Two-way (genotype × sex) ANOVA indicated that there were no genotype differences in the total movement distance (A), center distance (B), time spent in the center (C), and time spend in the outer zoon (D). The distance and duration in center arena for females showed decreased tendency ($P<0.05$) compared with the control group, the periphery time were no difference between *Fry* conditional KO mice with the control group (both sexes). No difference was detected between genotypes. Data presented as mean ± SEM. *$P< 0.05$. Mfry versus WT.

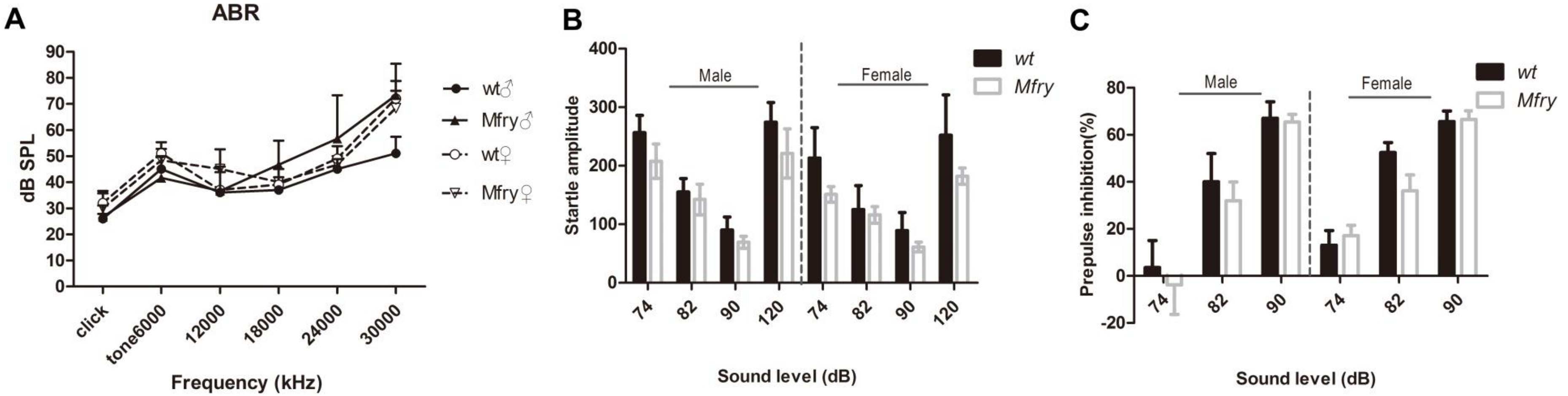

Supplementary Figures 3. (A) ABR, Auditory brainstem response test hearing sensitivity. 14 weeks old mice (n=5 for male and female WT mice, n=3 for male and female Mfry mice) were used. Mean ABR thresholds for the Mfry mice and WT mice (both sexes) showed no difference. **(B, C) PPI**. Male and female *Fry* conditional KO mice show no difference in PPI test. The responses to different levels of acoustic startle are presented in (B). The values of PPI% are presented in (C). Three-way (genotype × sex × sound level) ANOVA with repeated measures indicated no significant difference in acoustic startle response and PPI% between genotypes. Data presented as mean ± SEM. Mfry versus WT.

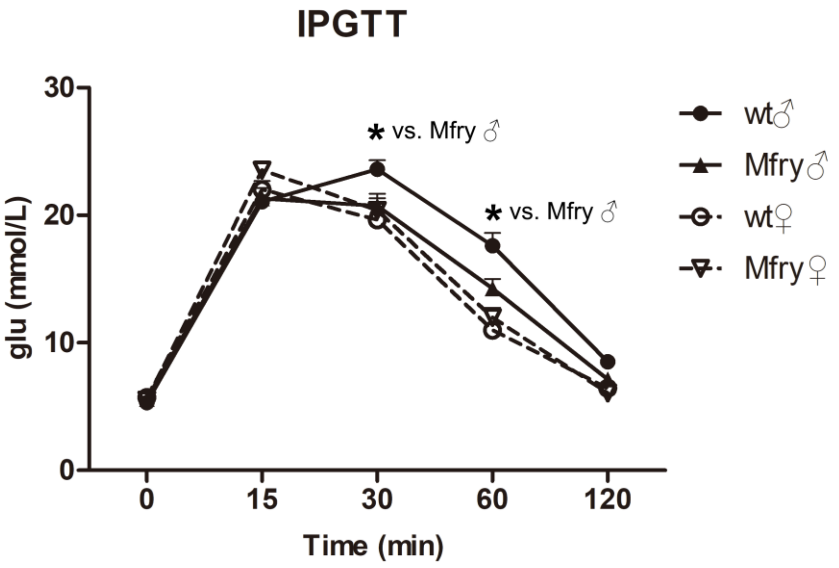

Supplementary Figures 4. Intraperitoneal glucose tolerance test (IPGTT) in the Mfry mice and WT mice (both sexes). IPGTT (2 g/kg glucose challenge, i.p.) curve depicting glucose clearance capacity. 13 weeks old mice (n=5 for male and female WT mice, n=7 for male and female Mfry mice) were used. The glucose metabolism in male *Fry* KO mice is faster than in WT mice after 30 min to 60 min glucose injection. However, in female Mfry mice, blood glucose level showed no difference than WT female mice. Data presented as mean ± SEM. Statistical significance: *P< 0.05. Mfry versus WT.

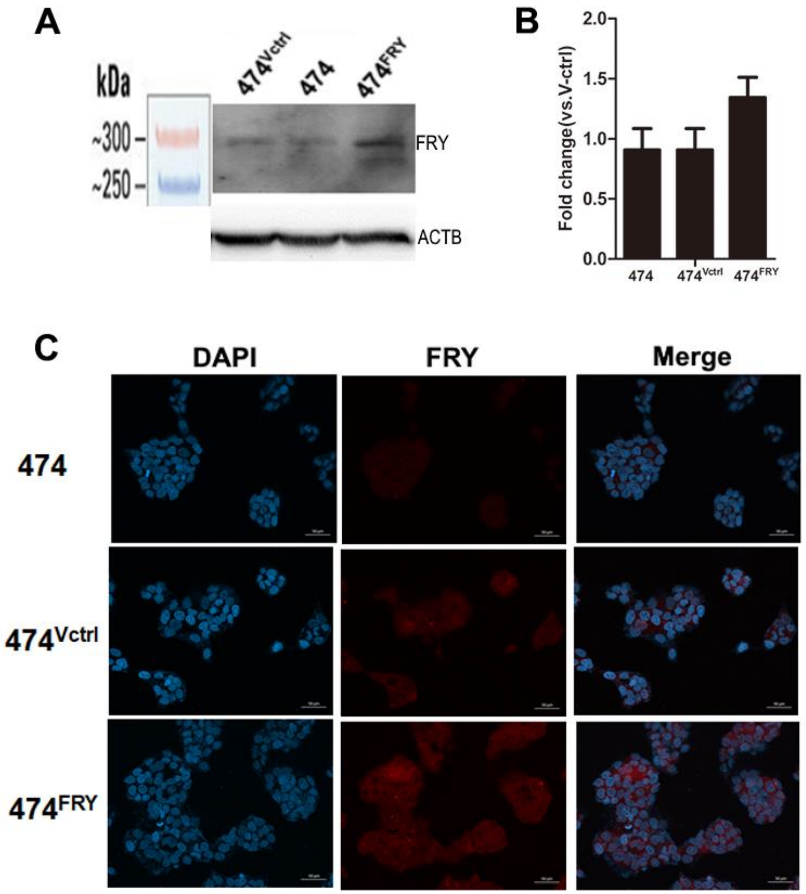

Supplementary Figures 5. FRY's overexpression in BT474. (**A, B**) A representative western blot analysis shows the increased ectopic FRY protein levels in BT474 human breast cancer cells. Quantification of FRY level was performed by normalization to ACTB levels. (**C**) FRY and ACTB protein levels in 474^{Vctrl} and 474^{FRY} cells measured by cell immunofluorescence analysis (nuclei stained with DAPI). Bars represent the mean ± SEM of three independent experiments.

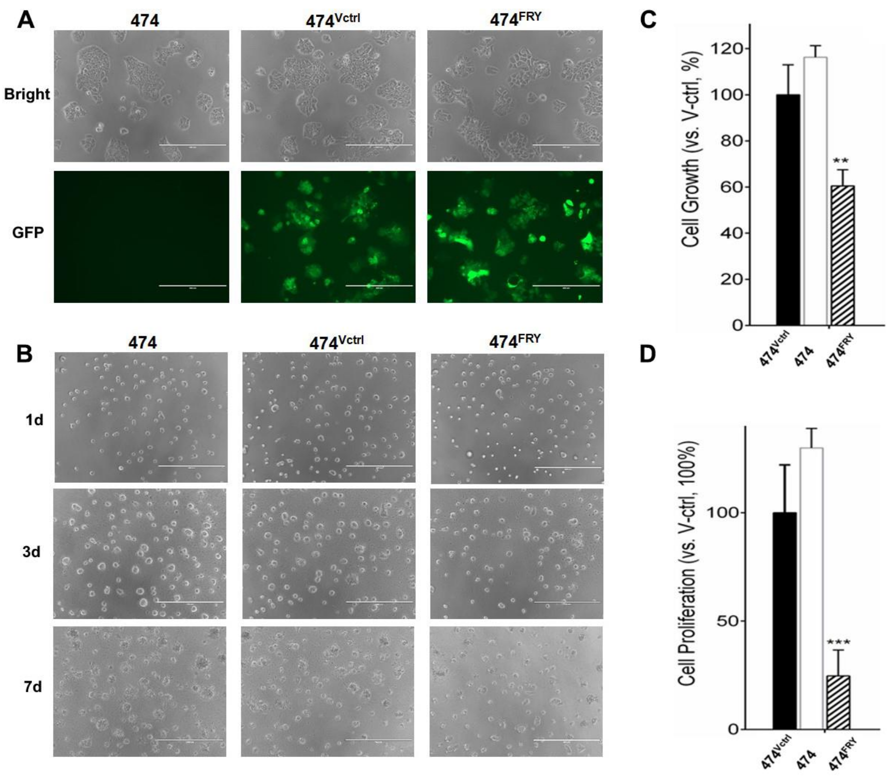

Supplementary Figures 6. Morphology and suppresses growth and proliferation of BT474 breast cancer cells with ectopic FRY. (**A**) Representative image of cell morphology in monolayer culture. (**B**) Representative image of cell morphology in 3D Matrigel culture. (**C, D**) Cell growth and proliferation of 24 hours were evaluated with MTT assay and BrdU assay. Bars represent the mean ± SEM of three independent experiments. Statistical significance: ** $P<0.01$; *** $P<0.001$.

Supplementary Table 1. Summary of RNA-seq sequencing results.

		Total	Uniquely Mapped		Multiple Mapped	
MDA-MB-231	**231^{PRT}-R1**	37,903,096	32,361,910	85.38%	4,973,638	13.12%
	**231^{PRT}-R2**	40,926,882	34,249,761	83.69%	6,028,892	14.73%
	**231^{Vctrl}-R1**	28,801,886	24,017,111	83.39%	4,046,581	14.05%
	**231^{Vctrl}-R2**	26,440,342	22,190,953	83.93%	3,829,323	14.48%
	**231^{FRY}-R1**	29,983,356	25,363,273	84.59%	4,087,818	13.63%
	**231^{FRY}-R2**	38,246,967	32,378,265	84.66%	5,283,380	13.81%

Supplementary Table 2. The list of genes regulated by Hippo-Yap signaling pathways

geneId	geneName	231^{PRT}-R1	231^{PRT}-R2	231^{Vctrl}-R1	231^{Vctrl}-R2	231^{FRY}-R1	231^{FRY}-R2
ENSG00000007866.14	TEAD3	22.90804514	28.86666565	25.87544372	30.22216072	27.68416238	27.48920674
ENSG00000014138.4	POLA2	35.29814595	42.27514644	102.4419939	46.2713261	39.27459836	95.40920305
ENSG00000018408.10	WWTR1	60.75571069	52.95068847	15.29862001	68.07332611	59.21774162	13.20062171
ENSG00000019549.4	SNAI2	9.003285094	24.45766679	0.209857613	19.63197202	18.00261531	0.041446222
ENSG00000020922.8	MRE11A	27.90359831	35.27199087	17.68050392	31.23936134	34.7818543	21.30335811
ENSG00000070950.5	RAD18	21.22404511	35.72036363	15.56094203	30.87769001	42.17484394	18.48501501
ENSG00000072571.15	HMMR	7.742637024	13.19496995	29.12823673	11.29092691	16.2730143	27.41667585
ENSG00000072682.14	P4HA2	68.99696225	35.25063978	15.05728375	41.37746088	28.23257245	19.84237878
ENSG00000073282.8	TP63	1.561698355	0.427021681	1.678860906	0.248649041	0.590595464	1.885803101
ENSG00000074800.9	ENO1	2729.246624	2791.451406	976.8137397	3260.139297	2701.446931	954.3614309
ENSG00000078401.6	EDN1	10.01932981	19.33340662	3.683001113	14.50075999	37.39734849	1.885803101
ENSG00000078900.10	TP73	1.091307284	2.914422974	0.293800659	1.05110731	3.163904272	0.165784888
ENSG00000081237.14	PTPRC	0	0	0	0.056511146	0	0
ENSG00000089685.10	BIRC5	109.1307284	132.8357695	51.10032884	129.9417284	130.2052071	45.07276643
ENSG00000094804.5	CDC6	72.24266064	103.7342419	47.43831349	101.1436486	103.438577	44.16094954
ENSG00000095752.2	IL11	54.6970737	108.4848581	0.31478642	104.8846864	97.22677827	0.186507999
ENSG00000099860.4	GADD45B	23.95231332	47.33535336	15.33009865	45.81923693	54.5140706	13.7601457
ENSG00000100311.12	PDGFB	0.357497214	0.918096615	14.64806141	1.299756351	0.854254153	14.56834703
ENSG00000101003.8	GINS1	27.5461011	32.27216356	28.3832422	33.27376259	29.73015381	30.79454295
ENSG00000101665.4	SMAD7	6.707776668	7.633012552	9.16028482	8.725320896	6.053603507	10.66204061
ENSG00000102265.7	TIMP1	276.9850781	172.3673016	2.854063541	240.4662272	172.7808123	2.683642875
ENSG00000102554.9	KLF5	11.43991084	9.960280714	20.47161018	8.239325043	12.06502162	26.18365075
ENSG00000103647.8	CORO2B	3.217474924	10.48338227	0	11.06488233	10.67290374	0.041446222
ENSG00000105810.5	CDK6	11.77859241	23.3687615	4.952639674	15.21280042	20.91340724	6.817903519

ENSG00000107485.11	GATA3	5.193117421	3.127933815	59.95632012	2.170027995	2.119815862	62.84283411
ENSG00000107551.16	RASSF4	20.28326297	7.227341955	0.178378971	11.70910939	7.403535996	0.279761999
ENSG00000108055.9	SMC3	58.55428048	84.82785697	22.11899244	51.741605	85.33049821	19.70767856
ENSG00000109321.6	AREG	75.77059367	7.141937618	46.3470539	19.99364335	4.566568499	43.85010288
ENSG00000111602.7	TIMELESS	44.00978858	43.28932293	70.62757976	44.59859618	46.79414418	77.54588136
ENSG00000111704.6	NANOG	0	0	0	0.022604458	0	0.031084667
ENSG00000112118.13	MCM3	207.8658142	314.0424199	156.2599789	327.7533428	337.8416981	161.308696
ENSG00000113520.6	IL4	0.348089392	0.256213009	0	0	0.274205037	0
ENSG00000113578.13	FGF1	1.298279355	6.319920882	0.419715227	9.20001452	15.2078332	0.113977111
ENSG00000114019.10	AMOTL2	62.09162133	80.29075161	41.10061356	81.01437847	73.8666184	41.64309156
ENSG00000115008.5	IL1A	6.416134205	16.2054728	0	7.742026961	3.174450619	0
ENSG00000115414.14	FN1	377.3665326	56.63375047	5.005104077	77.79324317	52.51026456	7.657189515
ENSG00000115935.12	WIPF1	22.91745297	26.85966375	0.178378971	19.47374081	23.24415005	0.321208221
ENSG00000117394.15	SLC2A1	192.3146854	166.228865	78.12998943	165.6228658	136.5224693	80.07410091
ENSG00000117724.8	CENPF	62.09162133	106.9689311	52.92609007	66.29887613	107.4461891	47.48700886
ENSG00000118193.7	KIF14	13.25562037	25.29035907	4.931653913	14.1164842	26.88263996	5.771386414
ENSG00000118523.5	CTGF	71.31128632	184.4840418	0.346265062	192.4769622	208.6594867	0.41446222
ENSG00000122691.8	TWIST1	0	0	19.78957293	0	0	18.64043834
ENSG00000123610.3	TNFAIP6	0	0.149457588	0	0	0	0
ENSG00000124216.3	SNAI1	1.909787747	0.523101559	0.776473169	0.892876102	0.548410074	1.150132661
ENSG00000124813.16	RUNX2	17.78078247	15.58629136	0.356757943	13.77741732	11.70644581	0.694224219
ENSG00000125398.5	SOX9	29.11720728	36.94805097	12.82230017	41.52438986	33.0311606	13.68761482
ENSG00000125482.8	TTF1	12.19253655	15.16994523	31.47864199	13.49486159	15.07073068	31.7374445
ENSG00000125538.7	IL1B	13.94239134	29.0054477	0	33.34157596	12.01228989	0
ENSG00000125845.6	BMP2	0.084670393	0.074728794	0	0.847667185	0	0
ENSG00000127129.5	EDN2	2.474257032	1.270389502	4.060744817	0.712040436	0.369122165	3.253528427

ENSG00000129474.11	AJUBA	99.19606899	94.53192468	30.57625426	98.9849228	99.20949162	31.69599828
ENSG00000129757.8	CDKN1C	10.56498345	4.526429821	0.734501647	7.662911357	4.081436511	0.891093773
ENSG00000129810.10	SGOL1	21.36516243	29.64598022	16.59973721	23.47472992	32.3878334	16.08113414
ENSG00000130592.9	LSP1	0	0.042702168	1.25914568	0.214742354	0.094917128	0
ENSG00000131470.10	PSMC3IP	32.71099506	45.73402206	19.67415125	35.87327529	42.65997593	13.79123037
ENSG00000131747.10	TOP2A	255.9303738	329.1483119	151.7375473	225.8863516	395.5302194	156.5734652
ENSG00000132170.15	PPARG	31.21515145	23.12322404	4.459474283	35.00300364	27.58924525	2.476411765
ENSG00000134333.9	LDHA	2657.502577	2409.576592	704.1982072	3060.3837	2005.577825	682.5881917
ENSG00000134690.6	CDCA8	66.57915215	89.1514515	39.04400895	84.55197619	99.23058431	38.38956313
ENSG00000134762.12	DSC3	0	0	0.031478642	0	0	0.051807778
ENSG00000134954.10	ETS1	69.59906282	185.0925477	0.104928807	178.6430338	152.2787126	0.269400443
ENSG00000135903.14	PAX3	0	0	0	0	0	0
ENSG00000136244.7	IL6	99.49711928	18.84233168	0	23.40691655	11.02093321	0
ENSG00000136634.5	IL10	0.112893857	0	0	0	0	0
ENSG00000136997.10	MYC	85.2724933	115.6267957	97.66773323	143.5609145	106.9399644	109.2315181
ENSG00000137440.3	FGFBP1	0.037631286	0	1.017809424	0.079115604	0	1.253748216
ENSG00000137462.6	TLR2	5.672916313	2.882396348	0.178378971	2.576908244	0.421853903	0.50771622
ENSG00000137807.9	KIF23	62.06339787	91.820337	26.85128162	73.01240024	105.5478465	33.31240093
ENSG00000138182.10	KIF20B	20.4714194	31.04447622	9.097327537	15.31452048	36.57473338	9.41865395
ENSG00000140464.15	PML	43.21953158	37.56723241	7.491916795	39.42217524	32.48275052	7.740081959
ENSG00000142279.8	WTIP	8.523486201	9.138263978	1.028302305	7.61770244	14.94417451	1.564594881
ENSG00000142871.11	CYR61	618.9217552	771.8416888	10.19908001	788.7825716	666.6346301	10.43408639
ENSG00000142949.12	PTPRF	115.4621922	79.85305439	115.3377443	90.06746401	65.57718921	113.7077101
ENSG00000143514.12	TP53BP2	28.35517374	34.40727196	12.00385548	33.74845621	31.5019402	11.09722594
ENSG00000145386.5	CCNA2	128.3509076	136.6362624	39.54766723	104.9072909	124.3730769	43.28021732
ENSG00000145632.10	PLK2	197.4795793	151.7635055	99.50398735	140.9840063	141.1101305	87.77273664

ENSG00000146670.5	CDCA5	107.1080468	143.5753648	117.6671638	145.31276	141.7956431	118.7745107
ENSG00000146674.10	IGFBP3	32.11830231	50.48463826	56.31529053	37.86246762	58.82752676	36.04785159
ENSG00000148229.8	POLE3	98.57515278	130.8714698	61.99193897	132.9481214	132.2406522	59.27845902
ENSG00000148516.17	ZEB1	16.49191094	28.68518144	0.062957284	19.53025195	38.14613917	0.186507999
ENSG00000148677.6	ANKRD1	293.0348214	179.1462708	0.94435926	225.8524449	219.2796587	1.326279104
ENSG00000150907.6	FOXO1	2.041497247	3.138609357	0.524644033	2.712534993	1.993259691	0.663139552
ENSG00000152256.9	PDK1	21.19582165	14.47603499	6.694457864	16.73860136	13.79462263	7.118388629
ENSG00000152455.11	SUV39H2	24.55441389	41.05813465	21.60484129	33.73715398	42.10101951	25.64484986
ENSG00000154721.10	JAM2	0	0.064053252	0.094435926	0.067813375	0.073824433	0.031084667
ENSG00000157557.7	ETS2	18.46755344	21.24432864	2.738641854	20.95433282	22.34771051	2.642196653
ENSG00000158528.7	PPP1R9A	2.257877139	1.25971396	4.879189509	1.604916538	1.12845919	4.901015752
ENSG00000159216.14	RUNX1	28.0729391	30.82028984	19.19147874	35.70374185	37.75592431	23.23060743
ENSG00000160255.12	ITGB2	19.92576576	4.825344998	2.035618849	9.968566101	5.789944817	1.45061777
ENSG00000161638.6	ITGA5	77.30406856	56.72983035	0.870909095	80.70921828	43.01855175	0.663139552
ENSG00000162772.12	ATF3	6.293832526	6.394649676	5.718619962	7.063893212	4.250178072	4.165345311
ENSG00000163739.4	CXCL1	367.3283871	246.6583986	2.948499467	164.3231095	70.61834335	3.460759537
ENSG00000163930.5	BAP1	83.22158823	104.8658494	43.33559715	118.0517834	103.5018551	43.13515555
ENSG00000164035.5	EMCN	0.018815643	0	0.230843375	0	0	0
ENSG00000164045.7	CDC25A	21.00766522	33.13688246	10.71323116	31.60103267	37.3762558	12.39242038
ENSG00000165025.10	SYK	0	0	6.883329716	0.011302229	0.010546348	7.04585774
ENSG00000165556.9	CDX2	0	0	0.115421687	0	0	0.538800886
ENSG00000166508.13	MCM7	267.2479829	338.7349486	317.420133	311.3538083	288.7906356	323.4670396
ENSG00000166582.5	CENPV	40.22784437	29.08017649	7.187623255	23.0791519	30.58440796	9.94709328
ENSG00000166949.11	SMAD3	79.66543174	72.42287713	7.407973749	76.45958013	63.27808544	8.651898843
ENSG00000167074.10	TEF	4.007731923	3.672386458	9.46457836	4.407869364	3.005709058	7.263450406
ENSG00000167601.7	AXL	240.2757589	467.9196827	0.104928807	437.1024097	459.9789494	0.310846665

ENSG00000167900.7	TK1	217.9133674	234.7551693	83.94304532	246.2755729	246.9216357	79.01722224
ENSG00000168036.12	CTNNB1	219.3809876	271.3616029	91.85467734	309.7262873	265.6941344	89.27516219
ENSG00000169554.12	ZEB2	4.177072708	4.355621148	0	5.436372216	6.844579575	0
ENSG00000170365.5	SMAD1	7.027642597	7.761119056	16.92501651	7.990676002	5.705574037	15.98788014
ENSG00000170734.7	POLH	26.13492789	30.39326816	8.688105191	22.49143599	36.13178678	10.30974772
ENSG00000171552.8	BCL2L1	359.5951579	364.1747653	131.7695954	415.5829655	339.5607528	118.2460714
ENSG00000171791.10	BCL2	2.615374353	4.163461392	7.386987988	4.057500261	4.766949103	8.247798178
ENSG00000171848.9	RRM2	127.5324271	302.7690475	114.4773281	210.6396445	355.3591815	100.9837199
ENSG00000172260.9	NEGR1	1.345318462	7.590310384	0	5.052096425	12.06502162	0.020723111
ENSG00000174442.7	ZWILCH	35.28873813	50.92233549	26.62043825	39.37696632	47.70113007	26.8467903
ENSG00000175387.11	SMAD2	48.65725236	38.51735565	64.46825881	43.5587911	44.41066963	64.71827565
ENSG00000175592.4	FOSL1	272.2059048	477.4742929	0.220350494	548.2259266	446.342522	0.176146444
ENSG00000176171.7	BNIP3	417.9800976	230.7091388	47.72162126	313.3430007	213.5002603	43.90191065
ENSG00000178568.9	ERBB4	0.084670393	0.010675542	1.689353787	0	0	3.077381984
ENSG00000181449.2	SOX2	0.677363142	0.085404336	0.545629795	0.113022291	0.137102518	1.005070884
ENSG00000182197.6	EXT1	173.0098358	226.7591883	20.40865289	244.5463319	233.2113839	18.97200812
ENSG00000182866.12	LCK	0	0	0	0.056511146	0.084370781	0
ENSG00000183207.8	RUVBL2	155.0126734	270.9132301	73.66002227	264.4947663	281.7245827	79.60783091
ENSG00000183527.7	PSMG1	93.78657168	125.4269433	83.20854367	132.2812898	135.1408978	84.45703888
ENSG00000184445.7	KNTC1	50.51059317	46.79090072	69.36843408	48.2492162	59.78724439	63.01898055
ENSG00000185697.12	MYBL1	20.46201158	39.91585165	10.0416868	18.29830898	46.61485627	9.263230617
ENSG00000186575.13	NF2	3.876022423	4.910749334	11.87794091	5.77543909	5.800491165	11.21120305
ENSG00000187079.10	TEAD1	60.51110734	72.80719665	22.89546561	61.6310555	64.82839853	25.06460275
ENSG00000196230.8	TUBB	1965.293893	2430.607409	1112.308308	2333.876411	2599.010257	1150.505677
ENSG00000196470.7	SIAH1	11.21412313	12.7252461	25.64460035	13.61918611	13.0352856	25.68629608
ENSG00000196611.4	MMP1	1.975642497	12.27687334	0.031478642	11.04227787	7.519545819	0.020723111

ENSG00000197905.4	TEAD4	17.54558694	41.57056067	26.08530133	46.50867291	42.3857709	24.80556387
ENSG00000204531.11	POU5F1	1.006636891	0.854043362	0.304293539	0.565111457	0.727697983	0.942901551
ENSG00000214049.6	UCA1	22.21186636	9.0421841	11.37428264	7.538586836	16.19918987	11.7810886
ENSG00000249915.3	PDCD6	114.0886503	107.3852773	567.3815362	110.6714277	95.07532337	560.2493059
